# A rich and bountiful harvest: Key discoveries in plant cell biology

**DOI:** 10.1093/plcell/koab234

**Published:** 2021-09-15

**Authors:** Alice Y Cheung, Daniel J Cosgrove, Ikuko Hara-Nishimura, Gerd Jürgens, Clive Lloyd, David G Robinson, L Andrew Staehelin, Dolf Weijers

**Affiliations:** Department of Biochemistry and Molecular Biology, Molecular Cell Biology Program, Plant Biology Program, University of Massachusetts, Amherst, Massachusetts 01003, USA; Department of Biology, Penn State University, University Park, Pennsylvania 16802, USA; Faculty of Science and Engineering, Konan University, Kobe 658-8501, Japan; ZMBP-Developmental Genetics, University of Tuebingen, Tuebingen 72076, Germany; Department of Cell and Developmental Biology, John Innes Centre, Norwich NR4 7UH, UK; Centre for Organismal Studies, University of Heidelberg, Heidelberg D-69120, Germany; Department of Molecular, Cellular and Developmental Biology, University of Colorado, Boulder, Colorado 80309-0347, USA; Laboratory of Biochemistry, Wageningen University, Wageningen 6708WE, the Netherlands

## Abstract

The field of plant cell biology has a rich history of discovery, going back to Robert Hooke’s discovery of cells themselves. The development of microscopes and preparation techniques has allowed for the visualization of subcellular structures, and the use of protein biochemistry, genetics, and molecular biology has enabled the identification of proteins and mechanisms that regulate key cellular processes. In this review, seven senior plant cell biologists reflect on the development of this research field in the past decades, including the foundational contributions that their teams have made to our rich, current insights into cell biology. Topics covered include signaling and cell morphogenesis, membrane trafficking, cytokinesis, cytoskeletal regulation, and cell wall biology. In addition, these scientists illustrate the pathways to discovery in this exciting research field.

## Teaser

Seven senior plant cell biologists reflect on foundational contributions to a variety of topics, including pollen tube signaling, cell morphogenesis, membrane trafficking, cytokinesis, cytoskeletal regulation, and cell wall biology.

## Introduction

The concept of living organisms being composed of smaller units might have been unfathomable until Robert Hooke discovered small units, akin to monks’ cells, in cork. Since then, however, spurred by Antonie van Leeuwenhoek’s observation of living Spirogyra algae in pond water, cell theory has been central to biology. At its inception, cell biology was greatly influenced by discoveries made in plants, and plant cell biology has since been an active research field that has given birth to many exciting concepts and discoveries. Starting in the middle of the 20th century, new tools and methods have allowed researchers to explore sub-cellular processes and take “inventory of our acquired wealth,” as stated by Albert Claude in his Nobel Lecture in 1974. Plant cells not only share many features with the eukaryotic cells studied by Claude and others, but also have distinct structures and processes. In fact, new cellular structures and organelles are still being identified today, and we can rest assured that plant cell biology will continue to reveal the plant’s inner secrets. Given the wealth of discoveries on which our current textbooks are built, it would be unrealistic to exhaustively cover the major findings and highlight mechanisms and concepts across this broad field. Instead, in this review, seven senior plant cell biologists offer their personal reflections on the discoveries they witnessed in their respective domains within the field of plant cell biology. While these are personal retrospectives, these accounts illustrate what we did not know, what we have learned, and to some extent which questions still beg to answer.

## Alice Cheung: Journeying with the pollen tube to finding a versatile signaling module for reproduction and beyond

*Alice Cheung describes how, over the course of her career, her work has explored the nature of how cells grow at their tips, starting with pollen tubes and later including root hairs. Key findings are how small GTPases and actin-regulatory proteins coordinate the action of the actin cytoskeleton to support tip-focused growth. These works led to the identification of a cell-surface signaling module with broad functional implications for plant growth, reproduction, and coping with environmental challenges.*

**Figure 1:**
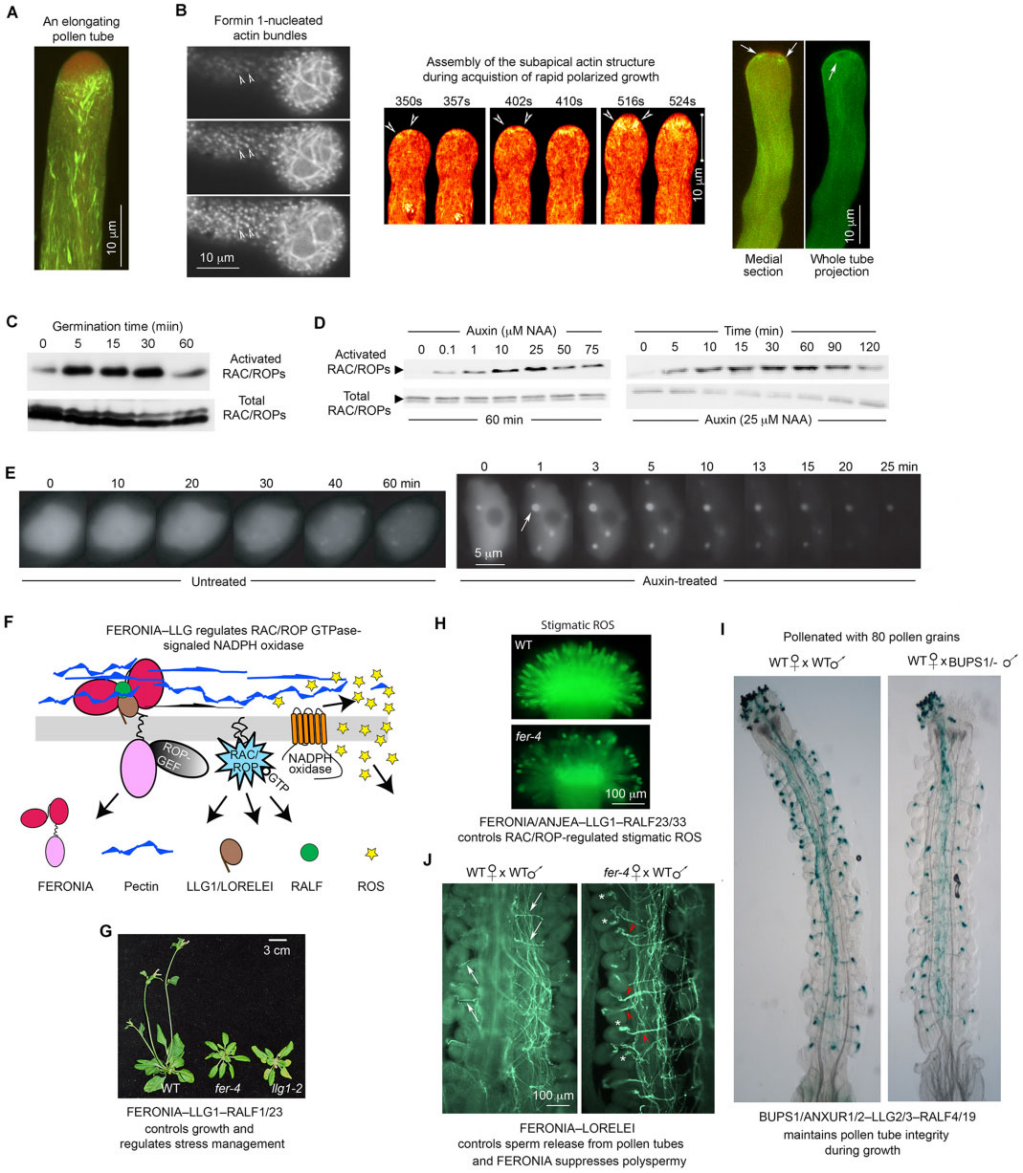
From the pollen tube to a cell surface signaling module with functions in diverse processes. A, Hallmark features in an elongating pollen tube: an apical vesicular zone (red), subapical actin (green/yellow) mesh, and long actin cables. B, Formin-regulated actin assembly. (Left) FORMIN HOMOLOGY PROTEIN1-stimulated assembly. Image shows three contiguous 1 µm optical sections showing bundles of actin (arrowhead) emanating from the cell membrane (top to bottom). (Middle and right) FORMIN HOMOLOGY PROTEIN5-generated subapical actin mesh (arrowheads). The middle panel shows the assembly of a subapical actin mesh in a pollen tube as it emerged from growth arrest to rapid polarized growth; arrowheads, nascent short actin bundles. The right panel highlights an annular configuration of the subapical actin structure (arrows). C and D, Protein pull-down assays showing that pollen hydration (C) and auxin (D) activate RAC/ROPs (upper panels). E, Nuclei of plant cells expressing IAA17-GFP showing rapid formation of nuclear protein bodies (arrow, as an example) in response to auxin. F, The FERONIA–LLG–RALF trimeric signaling module. G, Wild-type, *feronia*, and *llg1* plants showing the impacts of loss of the signaling module on growth and development. H–J, Functions of FER–LLG1–RALF-related signaling modules in reproduction. H, FERONIA-LLG1-RALF controls stigma ROS to regulate pollen germination. I, Pollen tube growth/integrity; image shows that only half the ovules were fertilized (reflected by blue dots) by pollen tubes from a heterozygous male parent (BUPS1/-). J, FERONIA controls multiple steps in pollen–pistil interactions: in wild-type pistils, single pollen tubes exit from the septum (arrows) and enter individual ovules to release sperm for fertilization. In *feronia* pistils, bundled pollen tubes exit from the septum (arrowheads) and enter into individual ovules; asterisks show pollen tube pile-up from nonruptured pollen tubes in mutant female gametophytes. Scale bars: 10 µm (A and B), 5 µm (E), 3 cm (F), 250 µm (I), and 100 µm (J). A from Cheung and Wu (2004)  Figure 6D; (B) adapted from Cheung and Wu (2004)  Figure 4A and Cheung et al. (2010) Supplemental Figure S5; (C) reprinted from Chen et al. (2013)  Figure 1A, with permission from Elsevier; (D) from Tao et al. (2002)  Figure 5C; (E) from Tao et al. (2005)  Figure 5, A and B; (F) modified from Duan et al. (2010)  Figure 3K and Li et al. (2015)  Figure 11; (G) modified from Li et al. (2015)  Figure 1C; (H) Adapted from Zhang et al., 2021a  Figure 7A; (I) from Ge et al. (2017) Supplemental Figure S6B, reprinted with permission from AAAS; (J) from Duan et al. (2020)  Figure 1B.

Lured by the inner beauty of a pollen tube, I entered the field of plant cell biology when live-cell imaging was becoming mainstream. Pollen tubes grow exclusively at their tips to deliver sperm for fertilization and have been a classical system for studying polarized growth. Growing pollen tubes maintain a dynamic and highly polarized cytoplasmic organization, with a mesh-like actin structure at the subapical region subtending an apical collection of vesicles and long actin cables in the distal organelle-rich cytoplasm (Figure 1A). Studies spanning decades performed by a large community of biologists have contributed to our understanding of the processes through which cell membrane and wall materials are carried to the elongating tip in vesicles that move along these actin cables (Cheung and Wu, 2008).

Our early cell biological studies helped establish small GTPases of the RHO (RAC/ROPs) and RAB families as important regulators of actin dynamics and vesicular trafficking in pollen tubes (Chen et al., 2002, 2003; Cheung et al., 2002; de Graaf et al., 2005). We pioneered the study of formins (actin-nucleating proteins that stimulate the assembly of linear actin) in plant cells and demonstrated the role of nascent actin assembly for pollen tube growth (Figure 1B;  Cheung and Wu, 2004; Cheung et al., 2010). These excursions added a cell biological context to our already ongoing molecular and biochemical work in male–female interactions (Wang et al., 1993; Cheung et al., 1995; Wu et al. 1995). We later incorporated root hairs, another tip-growing system, and seedlings into our studies. This merger has worked well because the seedling system allowed us to carry out fine mechanistic dissections, generating findings that provided hints to explore the considerably less accessible reproductive processes. Together, they led to the discoveries that pollen grain hydration (Figure 1C) and auxin treatment of seedlings (Figure 1D) both rapidly activate RAC/ROP GTPases (Tao et al., 2002; Chen, 2003, 2013; Wu et al., 2011). We also observed that auxin and RAC/ROPs mediate the assembly of nuclear protein bodies that comprise AUX/IAA repressors and components of the ubiquitin/26S proteasome apparatus, and they self-degrade the AUX/IAA repressors in response to auxin (Figure 1E). In hindsight, and given the emerging knowledge about ubiquitylation-dependent phase separation of the proteasome (e.g. Yasuda et al., 2018), it would be interesting to examine whether the auxin–RAC/ROP- and substrate-mediated processes for nuclear protein body formation are a manifestation of liquid–liquid phase transition. Given that RAC/ROPs are the major molecular switches that act along the inner cell membrane, we turned to searching for upstream regulators of RAC/ROPs to link signal perception to signal transduction.

Starting from using ROP-GEF1, a guanine nucleotide exchange factor that activates RAC/ROPs, as bait in yeast two-hybrid assays, we discovered the FERONIA receptor kinase as a surface regulator for RAC/ROPs (Duan et al., 2010). FERONIA functions with the glycosylphosphatidylinositol-anchored protein LLG1 (Li et al., 2015) as co-receptors on the cell surface (Figure 1F). FERONIA-LLG1 activates RAC/ROP-mediated reactive oxygen species (ROS) production by NADPH oxidase and intersects with multiple pathways to broadly affect growth and development. In reproduction, FERONIA and LORELEI, the ovule-specific LLG1 homolog, turn out to be critical for female fertility, since the FERONIA/LORELEI-to-ROS pathway is required for pollen tube bursting to release sperm in the female gametophyte, enabling fertilization (Figure 1J; Duan et al., 2014). We also showed that variants of the FERONIA–LORELEI coreceptor and ROS are important throughout pollination, controlling pollen germination on the stigma through RAC/ROP-mediated ROS production (Liu et al. 2021; Zhang et al., 2021a, 2021b) and preventing premature pollen tube bursting during the growth process (Ge et al., 2017, 2019; Figure 1, H and I).

FERONIA is a member of the malectin-like domain-containing receptor-like kinase family (Cheung and Wu, 2011; Doblas et al., 2018; Yang et al., 2021). A pioneering study of THESEUS1, which is closely related to FERONIA, showed that it functions as a sensor of cell wall perturbations to suppress the growth of cellulose-deficient seedlings (Hematy et al., 2007). We established biochemically that the extracellular domain of FERONIA binds pectin, a major cell wall polysaccharide (Feng et al., 2018). This property affects the pectic environment along the pollen tube growth pathway and affects how plants avert polyspermy (Duan et al., 2020; Figure 1J). The crucial roles of FERONIA in growth and development (Figure 1G;  Li et al., 2016), and discoveries from Michael Sussman’s and Cyril Zipfel’s laboratories that peptide regulators known as RAPID ALKALINIZATION FACTORS (RALFs) are ligands for FERONIA that regulate growth and immunity (Haruta et al., 2014; Stegmann et al., 2017) considerably expanded the field beyond reproduction. RALFs that regulate FERONIA and related receptor kinase-controlled processes in female tissues or pollen have also been identified (Ge et al., 2017, 2019; Liu et al., 2021).

How FERONIA–LLG1/LORELEI achieves its diverse functions remains unclear. Sensing multiple peptides as ligands, partnering with different LLG homologs, and utilizing the broadly functional RAC/ROPs and ubiquitous ROS as signaling mediators can all result in functional diversification. In seedlings, the FERONIA–pectin interaction has been linked to cell wall integrity; the loss of FERONIA renders seedlings more prone to salinity-induced cell bursting (Feng et al., 2018). The cell wall encounters myriad endogenous and extracellular signals from neighboring cells and the environment. The ability of FERONIA to interact with the cell wall could also contribute to its diverse biological roles. Sorting out how the FERONIA–LLG1 signaling module achieves its broad and profound functions in growth and survival will be a formidable challenge to tackle.

While working on two developmentally distant systems sometimes appeared chaotic, it has turned out to be immensely rewarding. Following logical next steps, even when it meant taking time to start from scratch so we could venture into unfamiliar territory, has helped us build perspectives. Discovering FERONIA as an upstream regulator of RAC/ROPs in seedlings and that FERONIA turns out to be a major regulator of female fertility (Escobar-Restrepo et al., 2007) was a lucky coincidence. It brought us back from pollen tube cell biology to where we started in male–female interactions and stimulated many exciting findings from many groups. *The Plant Cell* is home to many of the studies cited here. Elucidating the huge signaling capacity harnessed in large numbers of potential variants of the FERONIA–LLG–RALF signaling complex assembled from related proteins (Yang et al., 2021), their mechanisms, and their molecular interactions at high resolution will likely remain rich grounds for contributions to *The Plant Cell* in years to come.

## Daniel J. Cosgrove: Stress relaxation, expansins, and cell wall structure

*Daniel Cosgrove’s work has focused on understanding the complex biology and biochemistry that underlies the properties of the growing cell wall. A major focus of his work has been toward* *characterizing the contribution of cell wall loosening to cell growth. His career spans from classical physiological studies, through molecular identification of the expansin protein family, to modern modeling approaches.*

Irreversible cell enlargement (growth) requires concomitant water uptake and wall yielding. Decades of conflicting opinions about how these two physically distinct processes are causally connected went unresolved until Ray et al. (1972) clarified that wall “stress relaxation is the primary event in cell enlargement, whereas water uptake, volume increase, and extension (strain) of the cell wall are secondary.” They noted that “explicit treatment of the stress relaxation process…seems not to have been given in the plant growth literature.” Although stress relaxation was measured empirically in isolated walls (Yamamoto et al., 1970; Cleland and Haughton, 1971), how it instigated water uptake and growth in vivo was clarified only later by a new theory combined with experimental methods to measure stress relaxation in vivo (Cosgrove, 1985, 1987a). The principle of the method was to prevent cell water uptake and to measure the ensuing decay in turgor pressure as the wall relaxed. This work, along with related studies, provided the framework to determine which biophysical parameters control cell growth. In theory, either water uptake or wall yielding could limit cell growth, or the two processes could co-limit growth. A review of this field concluded that “reduction in cell turgor pressure, as a consequence of wall relaxation, serves as the major initiator and control point for plant cell enlargement” (Cosgrove, 1993).

Perhaps, you have seen the cartoon of a theoretician scrawling equations on a blackboard, leading to the punchline “Then a miracle occurs!” To some biologists, stress relaxation may seem a bit like that. To put a molecular face on wall stress relaxation, we may ask: what is it, what causes it, and how is it controlled? Physically, wall stress relaxation is a reduction in the tensile forces transmitted between polymers in the wall. This may originate with the cutting of load-bearing tethers within the wall, viscoelastic movements of wall polymers toward a lower energy state, or the release of noncovalent bonding between wall components. A satisfying answer to this question calls for a molecular model of the cell wall that includes the dynamic interactions of wall polysaccharides and their responses to forces, that is, a molecular model of wall mechanics.

The discovery of expansins opened one avenue to investigating this “miracle.” For many years, acid pH was known to stimulate wall expansion (dubbed “acid growth”), leading to the speculation that pH-sensitive hydrolases cut load-bearing crosslinks in the cell wall. Acid growth is best known in connection with auxin-stimulated growth (Rayle and Cleland, 1992; Du et al., 2020), but it is also connected to many other stimuli that modulate growth. A concerted hunt for the catalysts behind acid growth led us to discover α-expansins (EXPA; McQueen-Mason et al., 1992). These small proteins are present in growing cell walls in catalytic amounts and their activity is maximal at low pH (∼4) and minimal at neutral pH. The “trick” in identifying EXPAs was the use of a constant-force extensometer to measure cell wall creep (time-dependent, irreversible extension). Acid-induced creep in native cell walls was known to require endogenous proteins, potentially enzymes (Cosgrove, 1989). Contrary to the enzyme hypothesis, however, EXPAs lack enzymatic activity (Cosgrove, 2020). They rapidly induce wall creep without weakening the cell wall (McQueen-Mason and Cosgrove, 1995; Yuan et al., 2001).

**Figure 2:**
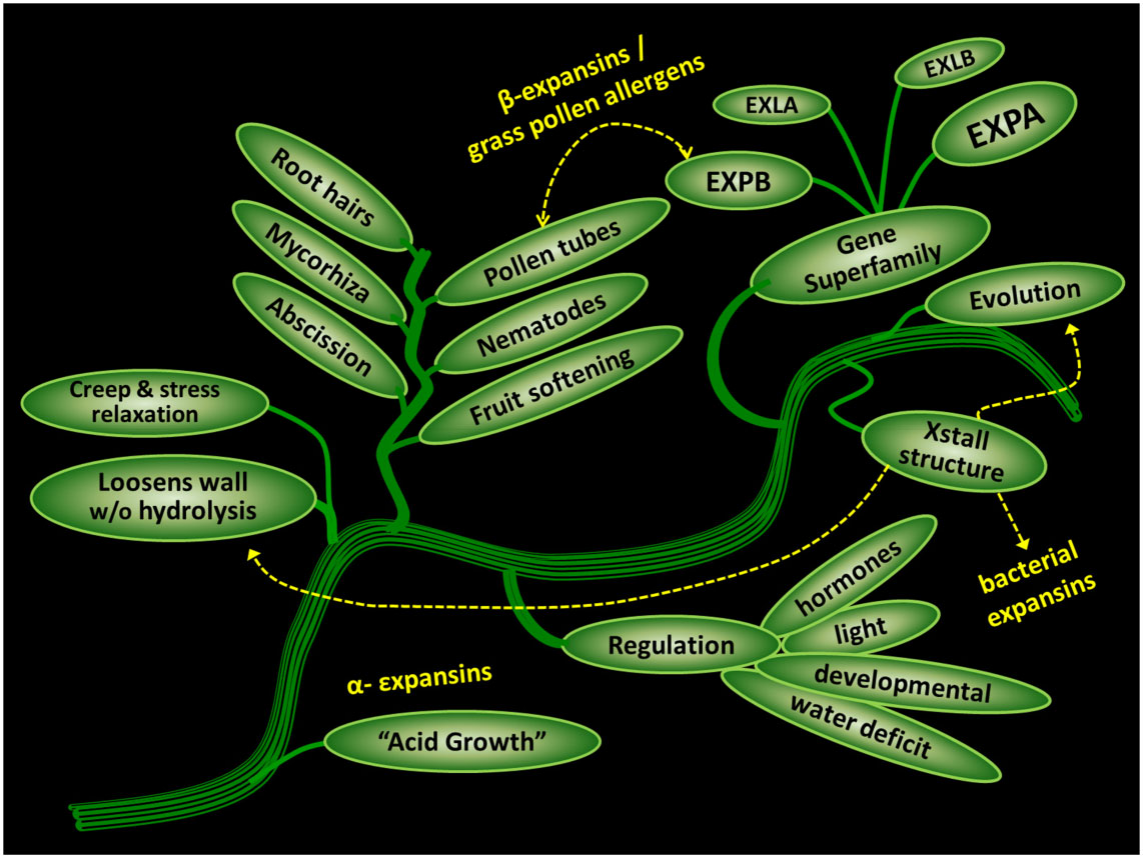
Evolution of expansin-related research topics. The figure depicts the development of the expansin research field, starting with the discovery of EXPA as the mediator of acid growth to its involvement in many developmental processes, the discovery of EXPBs with a special function in grass pollination biology, the evolution of expansins as a multigene family, and the discovery of bacterial expansins by way of protein crystallography. Image copyrigth Daniel J. Cosgrove, used with permission.

After the cloning of EXPA (Shcherban et al., 1995), it became apparent that expansins in land plants comprise a multigene superfamily, consisting of two moderately large families, EXPAs and β-expansins (EXPBs), and two smaller groups (EXLA and EXLB; Sampedro and Cosgrove, 2005). Differential expression of EXPA genes is connected with cell growth and many other developmental processes involving wall loosening (Figure 2). The role of EXPBs has not been studied in depth, except for an evolutionary outlier, a group of proteins expressed abundantly and specifically in grass pollen (Sampedro et al., 2015). Unlike EXPAs, these pollen EXPBs solubilize the dominant hemicellulose in grass cell walls, arabinoxylans, but without lytic activity (Yennawar et al., 2006; Tabuchi et al., 2011; Wang et al., 2016). This action promotes penetration of growing pollen tubes between stylar cells (Valdivia et al., 2009). Much remains to be discovered about the physical actions and biological roles of the other EXPBs.

When the structure of the maize (*Zea mays*) pollen EXPB ZmEXPB1 was solved by X-ray crystallography (Yennawar et al., 2006), we learned it consists of two tightly packed domains with a long, flat surface suitable for polysaccharide binding. The N-terminal domain is homologous to family-45 endoglucanase, but it lacks the catalytic base normally needed for enzymatic activity. The C-terminal domain has a structure suggestive of a carbohydrate-binding domain. This structure led to the discovery of a structurally homologous protein from the bacterium *Bacillus subtilis* (Kerff et al., 2008) and thereafter the recognition that many phylogenetically diverse microbes encode expansins in their genomes, with many of these proteins likely involved in plant–microbe interactions (Nikolaidis et al., 2014; Narvaez-Barragan et al., 2020).

Although the structures of expansins are known in atomistic detail, how they induce wall stress relaxation and creep remains uncertain. Recent results from xyloglucanase digestions (Park and Cosgrove, 2012; Zhang et al., 2019) cast doubt on traditional depictions of cell walls in which cellulose microfibrils are connected together via xyloglucan or pectin tethers. Consequently, we recently took a fresh approach by combining coarse-grained modeling with mechanical assays to test the physical soundness of cell wall models (2021b). Single chains of cellulose, xyloglucan, and pectins were simulated with coarse-grained bead-and-spring models parameterized to correspond to the different physical properties of these wall polysaccharides. Multi-lamella walls were constructed from these model polymers, stretched in silico, and the stress–strain behaviors compared with real walls. The results: cellulose microfibrils spontaneously assembled into 2D networks that carried most of the (in-plane) tensile force, whereas pectin and xyloglucan transmitted a tiny proportion of the tensile force. Wall elasticity arose from straightening of the cellulose microfibrils, while plasticity originated from sliding between laterally bonded cellulose microfibrils. This model provided insights into the molecular bases of wall mechanics, but did not include the loosening action of expansins—a challenge for future research.

## Ikuko Hara-Nishimura: Endomembrane dynamics-based physiological functions of the endoplasmic reticulum and vacuoles

*Ikuko Hara-Nishimura’s work has focused on the dynamics of the endoplasmic reticulum (ER) and vacuole. Although the textbooks tell us that the ER passes proteins to the Golgi, in truth there are many different fates for proteins exiting the ER, some developmentally regulated and some stress responsive. Her work has identified functions for numerous specialized endomembrane compartments, and many regulators of their dynamics.*

**Figure 3:**
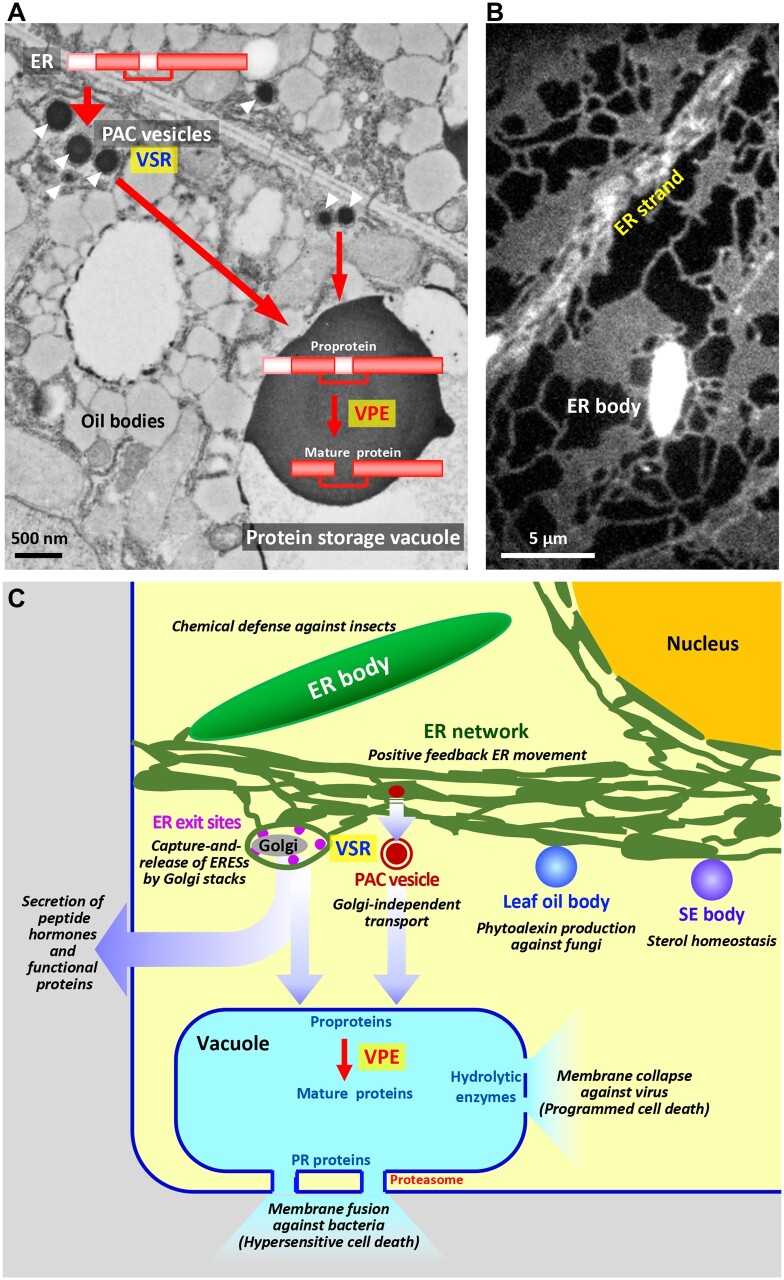
Endomembrane dynamics of the ER and vacuoles to support a variety of physiological functions. A, Ultrastructure of developing pumpkin seed cells showing PAC vesicles (arrows) responsible for Golgi-independent transport of storage protein precursors to protein storage vacuoles. VSR was discovered from isolated PAC vesicles. VPE converts proproteins to functional mature proteins in the vacuoles. B, GFP-labeled ER network of an *A. thaliana* cotyledon cell. ER strands rapidly stream along the longitudinal axis of the cell. The ER body is a large ER-derived organelle required for single-cell chemical defense in Brassicaceae plants. C, Endomembrane dynamics responsible for various physiological roles. The ER has the ability to generate distinct compartments with specialized functions. Vacuoles have the ability to remodel their membranes for defense against pathogen infection.

Here, I describe some of our discoveries of the endomembrane dynamics-based roles of the vacuole and ER. Before the era of the model plant *Arabidopsis thaliana*, vacuolar protein transport was investigated using developing seeds of a variety of plants, in which large amounts of storage proteins are synthesized on the ER and then transported to protein storage vacuoles. Analysis of pulse-chased developing pumpkin (*Cucurbita* sp.) seeds led us to discover a Golgi-independent pathway (Hara-Nishimura et al., 1985) in which proprotein precursors are delivered from the ER to the vacuoles via precursor-accumulating vesicles (PAC vesicles) (Figure 3A). PAC vesicles have >300 times the volume of COPII vesicles and enable efficient mass protein transport. They are generated under the stressed condition of excessive protein synthesis on the ER (discussed later). A membrane protein of PAC vesicles was later demonstrated to be a vacuolar sorting receptor (VSR; Shimada et al., 2002, 2003; Figure 3A).

Immediately after reaching the vacuole, proprotein precursors are converted into mature proteins. We biochemically isolated an enzyme responsible for the conversion from developing castor bean (*Ricinus communis*) and designated it vacuolar processing enzyme (VPE; Hara-Nishimura et al., 1993; Figure 3A). VPEs play various roles in the plant lifecycle including programmed cell death (Yamada et al., 2020a). We subsequently established *VPE/AEP*-knockout mouse and demonstrated that *VPE/AEP* is required for the maturation of lysosomal proteins (Shirahama-Noda et al., 2003). This is one of the rare cases in which plant studies provided insights into animal mechanisms. Many discoveries from *A. thaliana* studies have since contributed to our understanding of human biology and health (Jones et al., 2008).

We were fascinated with vacuolar membrane dynamics and discovered their association with defense strategies (Shimada et al., 2018a; Figure 3C). Viral infection triggers the vacuolar membrane to collapse in a VPE-dependent manner, resulting in the release of hydrolytic enzymes that prevent viral propagation in the cytosol (Hatsugai et al., 2004). On the other hand, bacterial infection causes the vacuolar membrane to fuse with the plasma membrane, resulting in the release of defense proteins that prevent bacteria from proliferating in extracellular spaces (Hatsugai et al., 2009). The discovery of these vacuolar protein relocations taught us that vacuoles are not necessarily the final destination or workplace of “vacuolar proteins.

The explosion of information about the model plant *A. thaliana* allowed us to significantly extend our knowledge about the functional peptides that are synthesized on the ER and then secreted to be used for the differentiation of target cells (Figure 3C). By focusing on *A. thaliana* genes encoding small proteins with signal peptides, we identified the peptide hormone stomagen, which positively regulates stomatal density in leaves, thereby increasing the efficiency of CO_2_ uptake (Sugano et al., 2010).

The development of imaging techniques has greatly extended our understanding of vesicle trafficking in plant cells (see also “Robinson’s” and “Staehelin’s” sections). A recent study combining variable-angle epifluorescence microscopy with high-temporal resolution imaging prompted us to develop a dynamic capture-and-release model of ER exit sites (ERESs) by Golgi stacks (Takagi et al., 2020). This model suggests that punctate ERESs wandering into an ER network cavity are captured by a Golgi stack for cargo transfer and are then released to reload with cargo and repeat the transfer (Figure 3C).

Furthermore, live cell imaging showed us that plant-specific myosin XI motors play critical roles in actin-myosin cytoskeleton-dependent dynamics of the endomembranes (see also “Cheung’s” section). We established multiple mutants of myosin XIs and elucidated their functions. First, myosin XI-I links the nuclear membrane to actin filaments and moves the nucleus in response to environmental stimuli (Tamura et al., 2013). Second, three myosin XIs (XI-K, XI-1, and XI-2) drive extensive streaming of the ER (Ueda et al., 2010; Figure 3B). Considering the fact that the ER has the largest endomembrane surface area in the cell, ER streaming might be the force driving cytoplasmic streaming, which has been a mystery since the late 18th century. Third, two myosin XIs (XI-F and XI-K) in elongating cells play roles in vigorous cytoplasmic streaming and organ straightening to adjust plant posture (Okamoto et al., 2015). These findings seem to reveal some linkage between endomembrane dynamics and organ movements.

Finally, I would like to emphasize that the ER has the ability to generate specialized compartments in response to stress (Figure 3C). As described above, PAC vesicles are generated from the ER in response to the abundant production of storage proteins (Figure 3A). Similarly, the abundant production of KDEL-ER-retention signal-tailed cysteine proteinases triggers the generation of specific organelles from the ER (Schmid et al., 1998; Okamoto et al., 2003). The largest ER-derived compartments in Brassicaceae plants are ER bodies (Figure 3, B and C). ER bodies establish a single-cell chemical defense in which damage from herbivore feeding causes ER-body β-glucosidases to react with glucosinolates and release repellent compounds (Yamada et al., 2020b). The question raised is how these organelles are generated from the ER in response to stress. We have recently succeeded in generating ER bodies in nonBrassicaceae plants including monocots by expressing two Brassicaceae-specific genes (*NAI2* and *BGLU23*) (Yamada et al., 2020b). This discovery could be used to produce herbivore-resistant crops.

Another question is how the sizes of ER-derived organelles are determined. An answer was provided from studies of oil bodies that are generated from the ER (Figure 3, A and C). Membrane proteins function in maintaining the proper sizes of oil bodies in seed cells, which preserves the viability of over-wintering oilseeds (Shimada et al., 2018b). We discovered two roles for oil bodies in leaves. First, oil bodies in fungal-infected and senescent leaves function as subcellular factories to produce antifungal compounds (Shimada et al., 2018b). Second, oil bodies that accumulate sterol esters (SEs bodies) are generated in response to the excess production of sterols (Shimada et al., 2019). SE bodies function in sterol homeostasis, in which excess sterols are converted to nontoxic sterol esters on the ER subdomains, which develop into SE bodies.

In conclusion, the ER and vacuole are flexible and dynamic endomembrane organelles. They and their membranes are remodeled in response to internal and external stresses. The ER has the ability to generate distinct compartments in response to the overproduction of materials, while vacuoles have the ability to remodel their membranes in response to pathogen infection. Their characteristic features might contribute to organelle engineering to create valuable crops.

## Gerd Jürgens: Membrane fusion in plant cytokinesis

*Gerd Jürgens entered plant cell biology through studying Arabidopsis embryogenesis, starting from genetic screens for patterning mutants. In addition to several key regulators of cell polarity and cell fate, he identified a wide range of genes involved in conserved cellular processes. Here he describes how this work uncovered key components of the machinery involved in membrane fusion, both during cytokinesis and in the secretory pathway.*

Cytokinesis marks the transition from one cell generation to the next by physically partitioning the dividing cell into two daughter cells. Although cytokinesis is a hallmark of all living systems, land plants (and most streptophytic algae) have evolved their own strategy—phragmoplast-assisted cell-plate formation—which contrasts with constriction from the surface, a mechanism shared by nonplant organisms with or without cell walls. Cell-plate formation was first described at the ultrastructural level in the 1960s (Hepler and Newcomb, 1967). However, modern functional analysis only started in the 1990s, with Arabidopsis playing a prominent role.

## The mutational beginning

**Figure 4:**
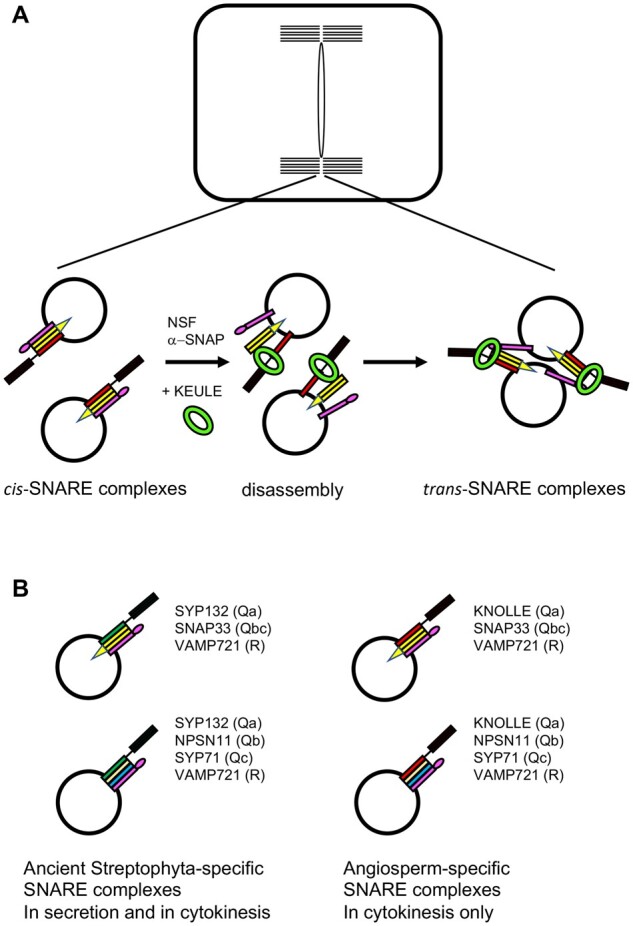
Membrane fusion during cytokinesis (model). A, Membrane vesicles arriving in the plane of cell division contain cis-SNARE complexes on their surfaces. Following disassembly, presumably by the AAA ATPase NSF and its co-factor α-SNAP, the now monomeric Qa-SNARE and R-SNARE proteins are captured by SEC1-related KEULE, which not only prevents reformation of the cis-SNARE complexes but also promotes *trans*-SNARE complex formation and thus the fusion of vesicles with each other and with the margin of the growing cell plate. Only complexes involving Qa-SNARE KNOLLE (green) and Qbc-SNARE SNAP33 (yellow) are depicted (see B, upper right). B, Evolutionary change of Qa-SNAREs involved in cytokinesis. The ancient SNARE complexes involving Qa-SNARE SYP132 appear conserved throughout the Streptophyta, mediating vesicle fusion during both secretion and cytokinesis. Cytokinesis-specific Qa-SNARE KNOLLE originated by gene duplication with the advent of flowering plants. The encoded protein retained the interaction with the SNARE partners of SYP132, forming two types of cytokinesis-specific SNARE complexes.

Our early research focused on the genetic analysis of embryo pattern formation. Among the many mutants isolated in a large-scale screen, we identified several alleles of two genes named *KNOLLE* (*KN*) (German for “tuber”) and *KEULE* (*KEU*) (German for “club”) that proved to play key roles in cell-plate formation (Mayer et al., 1991). In those days, getting hold of the relevant DNA sequence was a real feat: since there was no genome sequence to lean on, this analysis involved chromosome walking with yeast artificial chromosome (YAC) libraries and mapping of (hopefully) polymorphic YAC ends by genetic recombination relative to the mutation of interest. Eventually, the two genes were isolated and shown to encode a “syntaxin-related gene product” and “a SEC1 protein that binds the syntaxin KNOLLE,” respectively (Lukowitz et al., 1996; Assaad et al., 2001; Figure 4). The significance of these findings was not immediately clear to us, although the SNARE (short hand for soluble N-ethylmaleimide-sensitive-factor attachment receptor) hypothesis had just been formulated on the basis of biochemical studies in mammals and molecular analysis of yeast genes involved in protein secretion (Söllner et al., 1993; Ferro-Novick and Jahn, 1994). SNAREs are integral membrane proteins that form stable complexes of three or four SNARE proteins. These complexes contribute to the selective fusion of juxtaposed (endo)membranes such as membrane vesicles and their target membranes (heterotypic fusion) or two equivalent membranes (homotypic fusion; Jahn and Scheller, 2006). Different SNARE complexes are engaged in different membrane fusions, for example, the fusion of COPI vesicles with the ER or AP1 vesicles with the plasma membrane. Each SNARE complex comprises cognate members of different subfamilies named Qa, Qb, Qc, or R-SNAREs according to the amino acid residue in the center of their SNARE domain. In addition, the so-called Qbc-SNAREs have two SNARE domains. SNARE complexes comprise Qa, Qb, Qc, and R SNAREs or Qa, Qbc, and R-SNAREs. More importantly to us, the cell cycle-regulated accumulation of KNOLLE mRNA and protein, as well as the discovery of “unfused vesicles accumulating in the plane of cell division,” in *knolle* and *keule* mutant embryos and the absence of cytokinesis altogether in *knolle keule* double-mutant embryos hinted at the proteins’ roles in cell-plate formation (Lauber et al., 1997; Waizenegger et al., 2000).

## The boon and bane of genetic redundancy

Genetic analysis is a powerful approach for dissecting complex biological processes. In practice, however, related genes (paralogs) often functionally replace each other and thus mask deleterious mutational effects. Both *knolle* and *keule* mutant embryos gave rise to (abnormal) seedlings, although vesicle fusion during cytokinesis was disrupted from early on. The other source of frustration was our inability to identify KNOLLE-interacting SNARE partners based on *knolle*-like mutant phenotypes. A screen for yeast two-hybrid interactors pulled out Qbc-SNARE SNAP33. However, its knockout mutant only had a very mild cytokinesis defect and unrelated symptoms of cell death (Heese et al., 2001). At the same time, Qb-SNARE NPSN11 was identified as another KNOLLE interactor, and its knockout mutant was even viable (Zheng et al., 2002). The SNARE complexes known from yeast and mammals suggested that these two KNOLLE interactors might belong to different SNARE complexes or that the SNARE complex involved in cytokinesis might have an unusual composition. Eventually, this problem was solved. The *snap33 npsn11* double mutant displayed a strong *knolle*-like phenotype, including the accumulation of unfused vesicles in the division plane, and co-immunoprecipitation analysis revealed two KNOLLE-containing SNARE complexes, one comprising SNAP33 and the other NPSN11 (El Kasmi et al., 2013). Why KNOLLE forms two different SNARE complexes that both contribute to cell-plate formation is a mystery.

## The enlightening evolutionary perspective

KNOLLE redundancy was more difficult to unravel. A survey of SYP1 Qa-SNAREs revealed that SYP132 accumulated in all tissues and, together with KNOLLE, was the only SYP1 expressed in embryos (Enami et al., 2009). Moreover, the *knolle* mutant was rescued by expression of SYP132, but not SYP121 (aka PEN1), from the *KNOLLE* cis-regulatory sequences (Reichardt et al., 2011). Thus, SYP132 was the top candidate for a Qa-SNARE that shares a redundant function with KNOLLE in cytokinesis. This notion was further backed by the following evolutionary perspective: Cell division via cell-plate formation only arose in the streptophytic algae, while KNOLLE originated much later with the transition to flowering plants. The genome sequence of the filamentous charophyte green alga *Klebsormidium nitens* (aka *K. flaccidum*), which divides without cell-plate formation, had just become available (Hori et al., 2014). We identified single-copy genes encoding orthologs of SYP132 and SNARE partners of KNOLLE (SNAP33, NPSN11, SYP71, and VAMP721). Their Arabidopsis counterparts indeed formed complexes, and *syp132 knolle* embryos were multinucleate, resembling *knolle keule* double-mutant embryos (Park et al., 2018). The ancient SYP132 complexes were apparently supplemented with the KNOLLE complexes in flowering plants, most probably to meet the higher demand for membrane fusion in endosperm cellularization—a unique process associated with double fertilization in flowering plants.

The KNOLLE interactor KEULE plays a central role in trans-SNARE complex formation, mediating the fusion of membrane vesicles during cell-plate formation (Park et al., 2012). How KEULE might do so can be inferred from structural analysis of SEC1-related Vps33p, which functions in yeast vacuolar fusion, a topologically comparable process (Baker et al., 2015). KEULE also has counterparts with redundant functions that only originated during the evolution of flowering plants. Here, the paralog SEC1B plays a major role in secretion, leaving the lion’s share of the role in cytokinesis to KEULE, which is convincingly demonstrated in the cytokinesis phenotypes of *qa-snare sec1* double-mutant embryos (Karnahl et al., 2018).

## Other knowns and unknowns of membrane trafficking in cytokinesis

Cell-plate formation is a secretory process, with contributions from endocytosis (Reichardt et al., 2007). However, trafficking from the trans-Golgi network (TGN) to the division plane is entirely regulated by the late-secretory ADP-ribosylation factor guanine-nucleotide exchange factors (ARF-GEFs) BIG1-4 (Richter et al., 2014). Blocking secretory trafficking to the plane of cell division at different stations along the route revealed the formation of cis-SNARE complexes in the ER, which are only disassembled upon arrival at the division plane to form trans-SNARE complexes required for cell-plate formation (Karnahl et al., 2017). This disassembly requires N-ethylmaleimide-sensitive factor (NSF) ATPase activity which, however, has not been identified. The membrane vesicles that generate the cell plate pose another problem—are they homogeneous or are there KNOLLE-bearing and SYP132-bearing subpopulations? To answer this question, it might be necessary to isolate cytokinetic vesicles and determine their cargo—another daunting task.

## Clive Lloyd: Early MAPs—constructing dynamic microtubule arrays

*Clive Lloyd’s work has focused on the dynamic network of microtubules that mediate cellular processes. These dynamic elements of the cytoskeleton are constantly assembling and disassembling, and have crucial roles in mitosis. Microtubules* *are* *also reorient to direct plant growth and cell wall deposition. Here he describes the work of his group and others in identifying plant-specific microtubule-associated proteins (MAPs) that function to coordinate microtubule dynamics and cellular morphology.*

It is hard to think back to a time when microtubules were not dynamic. Electron microscopy studies on fixed, embedded cells had established that parallel groups of cortical microtubules co-aligned with cellulose microfibrils upon the plasma membrane. These microtubules could be in transverse, oblique, or longitudinal arrays that, with the onset of mitosis, bunched into a transient preprophase band (PPB), which forecast the alignment of the new cross-wall. It was inferred from these static images that cortical microtubules must reorientate—as was suspected when cells change the direction of expansion—but nothing was known about the mechanics nor of the MAPs that regulated behavior. Our only models were high and low molecular weight protein bands (e.g. MAP2 and tau) that co-purified with brain microtubules through cycles of assembly/disassembly (but let us not forget that this stringent method failed to detect the kinesin motor protein superfamily).

In the 1970s, I was investigating the cytoskeleton in animal cells and, when given the opportunity of forming a lab working on plant cells, decided on a dual approach: trying to identify plant-specific MAPs and applying immunofluorescence microscopy, which had already transformed the animal field. The significance of the latter approach was that it introduced a change in scale by allowing microtubule arrays to be seen in their unsectioned entirety (Lloyd et al., 1979). Then, in 1984, animal cell biology was revolutionized by studies using microinjected fluorescent tubulin showing that microtubules could dynamically interconvert between growing and shrinking states (Mitchison and Kirschner, 1984). By microinjecting neurotubulin into *Tradescantia* hairs, Peter Hepler’s group found that microtubules in immobile plant cells were, paradoxically, even more dynamic than in motile animal cells (Hush et al., 1994). And by microinjecting fluorescent brain tubulin into pea (*Pisum sativum*) epidermal cells, lab member Ming Yuan established that dynamicity extended to the whole cortical array when he saw cortical microtubules reorient through 90° in quite an unexpected way (Yuan et al., 1994).

In turn, microinjection was supplanted by green fluorescent protein (GFP) technology and, by using long-term time-lapse imaging of fluorescent fusion proteins, Jordi Chan (Chan et al., 2003; Lloyd and Chan, 2004) revealed how microtubule mosaics rotate around the outer face of Arabidopsis cells (Chan et al., 2007). Because microtubules guide the movement of cellulose synthase complexes along the plasma membrane (Paradez et al., 2006), microtubule rotation should also cause the synthase tracks to rotate, with implications for the layering of cellulose microfibrils in the cell wall. We confirmed that GFP-cellulose synthase 3 tracks also rotate, like the hands of a clock, and that this requires dynamic microtubules (Chan et al., 2010). These emergent properties of microtubule behavior—which cannot be intuited from reading the genome—are crucial to plant development. Adrian Sambade demonstrated this by selecting light-sensitive mutants in the DELLA/gibberellin signaling pathway and finding that light affects microtubule dynamics and their ability to switch growth axes (Sambade et al., 2012).

As far back as 1980, my colleague Toni Slabas demonstrated that presumptive but unidentified plant MAPs could be isolated by co-purification with brain microtubules, although mass identification of MAPs had to wait until lab members Edouard Pesquet and Paul Derbyshire used proteomics to identify >600 Arabidopsis MAPs (Derbyshire et al., 2015). Before the advent of proteomics, we isolated MAPs from detergent-extracted carrot (*Daucus carota*) cytoskeletons. In this way, Chan et al. (1999) purified a triplet of ca 65 kDa proteins that bundled brain microtubules in vitro, reproducing the characteristic 30 nm cross-bridges that maintained the parallelism of cortical microtubules in planta. These proved to be a new class of plant proteins, unrelated to classical brain MAPs (Smertenko et al., 2000). Because only one MAP65 isoform was expressed in carrot cells arrested in interphase, we suggested that other family members must attach to other microtubule arrays in dividing cells (Lloyd and Chan, 2004). Mutagenesis of the consensus phosphorylation site of cyclin-dependent kinase on Arabidopsis *GFP-AtMAP65-1* (of a nine-gene-member family) showed that labeling is downregulated at metaphase but returns to the central spindle at anaphase (Mao et al., 2005). It would appear that different MAP65 isoforms are expressed at different stages of the cell cycle and that the isoform that cross-links microtubules during interphase is switched off during metaphase by cell cycle-regulated phosphorylation.

**Figure 5:**
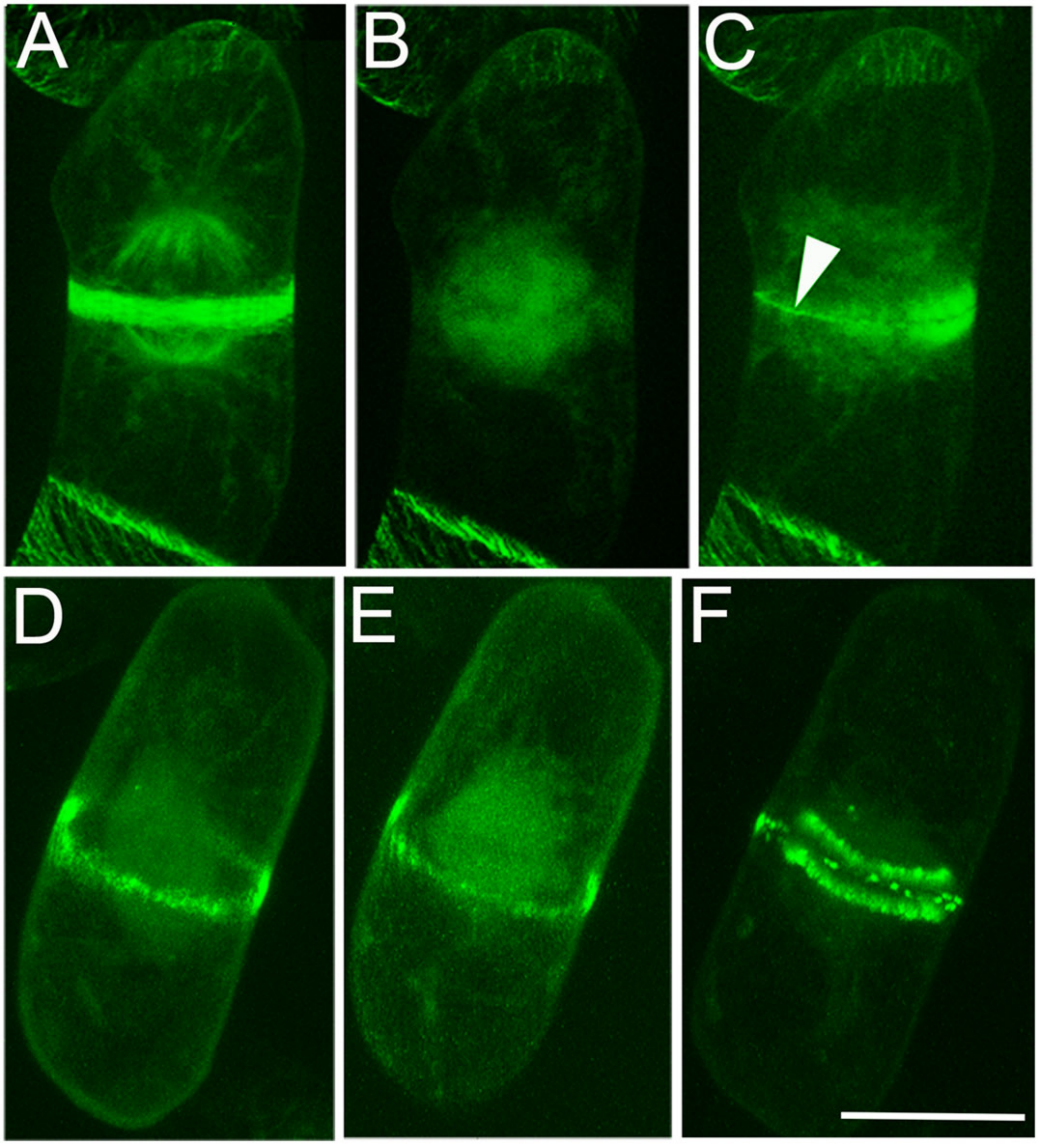
MAPs during mitosis. When expressed in tobacco BY-2 cells, GFP-AIR9 labels the PPB (A) but disappears from the cortex at metaphase (B). It reappears in a thin line at the cortex (arrowed in C) when the phragmoplast makes contact. In contrast, GFP-KCBP is detected in the PPB (D) but remains at the cortical division site in a particulate form during metaphase (E) and cytokinesis (F). Scale bar = 20 µm. Reproduced from Buschmann et al. (2015).

To screen for plant-specific MAPs that (1) would escape bioinformatic searches based on motifs from animal MAPs and (2) might not have bound to animal microtubules, we used Taxol to polymerize endogenous tubulin from Arabidopsis suspension cells. Co-purifying proteins were analyzed by mass spectrometry, yielding 55 candidates, amongst which was the five-member MAP70 family, found only in plants (Korolev et al., 2005). Another protein, occurring in plants and trypanosomes, was subsequently characterized by Buschmann et al. (2006). This 187 kDa protein, AIR9, decorates the PPB but then disappears during metaphase (Figure 5). However, AIR9 reappears as a ring at the predicted cortical division site the instant the phragmoplast makes contact, suggesting that AIR9 associates with a molecular imprint deposited by the PPB.

In searching for its binding partners, Henrik found that AIR9 interacts with the kinesin-like calmodulin-binding protein KCBP, a microtubule motor protein that stays in the cortical division site throughout mitosis (Buschmann et al., 2015; Figure 5). The ability of Arabidopsis KCBP to bind the cortex is conferred by its MyTH4-FERM domain, an evolutionarily conserved module known to link the actin cytoskeleton to microtubules. We hypothesized that minus-end-directed KCBP anchored at the former PPB site could “reel in” the plus ends of microtubules growing out from the phragmoplast. (KCBP’s connection with actin is also interesting, since actin filaments connect the dividing nucleus to the cortex [Traas et al., 1987])

Another positive marker of the cortical division site is TANGLED (Walker et al., 2007) and, in a recent collaboration with Henrik, Carolyn Rasmussen’s lab (Mir et al., 2018) made synthetic *air9 tan1* double mutants to establish that both proteins are required for normal division plane alignment. The longstanding suspicion that the PPB leaves behind an imprint—a molecular tidemark—has now been confirmed, and it would appear that AIR9, KCBP, and TAN1 are key parts of that complex.

## David Robinson: ER exit sites, ER import sites, and bidirectional transport in the early secretory pathway

*David Robinson’s work has focused on vesicle trafficking from the ER to the Golgi apparatus (anterograde pathway) as well as that from the Golgi to the ER (retrograde pathway). His group extended and expanded on studies carried out in yeast, and, in addition to describing key aspects of the plant secretory pathway, he identified several unique aspects. These include the findings that the plant Golgi apparatus is extraordinarily dynamic and that anterograde trafficking may occur at places where the ER and Golgi are in close proximity.*

I became fascinated with the plant Golgi apparatus since my days as a postdoc with Peter Ray (Stanford). After an interlude where I devoted my attention to the problem of microtubules and cellulose microfibril orientation, I returned to various aspects of the plant secretory pathway. These led to the first studies on endocytosis and clathrin-coated vesicle (CCV) isolation. The discovery of COP-coated vesicles in yeast and animals prompted us in the 1990s to ascertain their existence in plants. This is now indisputable. In the following section, I give a personalized account of the contribution of my group to the problems surrounding ER–Golgi vesicle-mediated cycling.

Studies on the plant Golgi apparatus were really given a boost when my colleague and friend, the late Chris Hawes, noted that the Golgi stacks of plant cells moved along the surface of tubular strands of ER (Boevink et al., 1998). However, even more interesting were the observations that this movement was intermittent (“stop-and-go”) and dependent on intact actin filaments. This immediately prompted the questions: where on the surface of the ER does ER export take place, and is export a continuous process or restricted to the stationary periods of the Golgi stacks? At that time, studies on animal and yeast cells had already established the existence and anterograde trafficking properties of COPII-coated vesicles, as well retrograde trafficking of COPI-coated vesicles (see Béthune and Wieland, 2018 for references). However, at the same time, antibodies against plant COPI and COPII-homolog proteins became available (Movafeghi et al., 1999), opening the way to the subcellular localization of COPI vesicles in plants (Pimpl et al., 2000).

My group next turned to the challenge of subcellular localization of COPII binding on the ER for anterograde transport, sites then termed ERES (ER exit sites). To this end, two experimental approaches were possible: immunofluorescence and the detection of transiently expressed fluorescently tagged [(X)-FP] COPII proteins. The first procedure applied to suspension-cultured tobacco BY-2 cells produced a uniform, punctate labeling of the ER, with the number of punctae greatly in excess of the number of visualized Golgi stacks (Yang et al., 2005). In contrast, daSilva et al. (2004) working with tobacco epidermal cells demonstrated a high degree of colocalization between the Golgi markers ST-GFP and ERD2-GFP and the GTPase SAR1-YFP (SAR1 is a GTPase that had previously been shown to be required for vesicle transport from the ER). This distribution pattern was later confirmed by examining YFP fusions to other COPII proteins (SEC23 and SEC24; Hanton et al., 2007).

**Figure 6:**
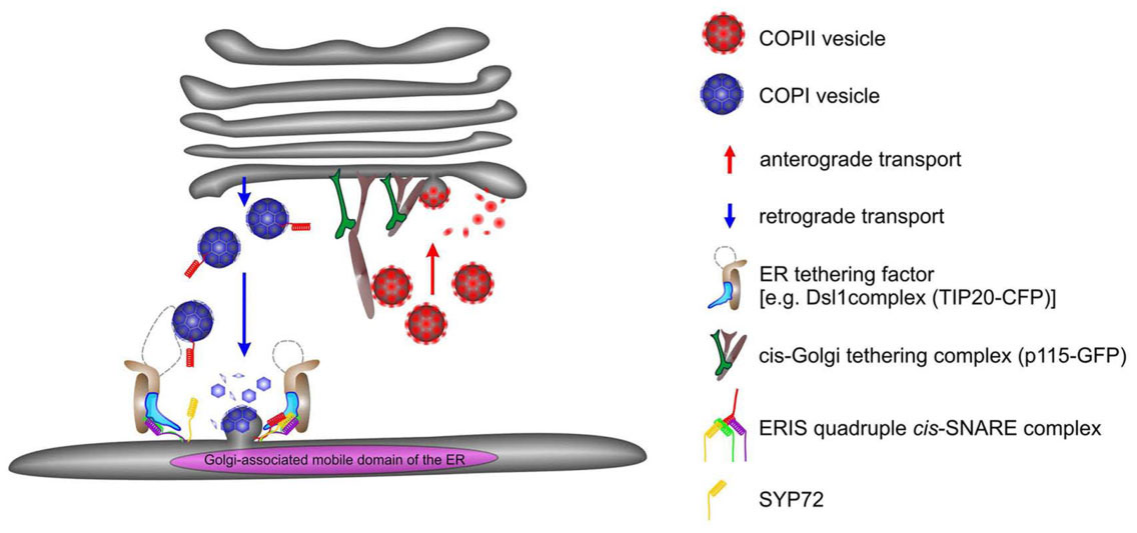
Cartoon portraying vesicle-mediated anterograde (COPII vesicles) and retrograde (COPI vesicles) between the ER and Golgi. Vesicle trafficking is accomplished by the sequential operation of long-range tethering complexes (in the case of retrograde trafficking to the ER, the complex includes the plant homolog to TIP20 of the yeast Dsl1 COPI-tethering complex, and in the case of anterograde trafficking to the Golgi, the cis-Golgi tethering complex includes the component p115), and short-range SNARE vesicle fusion complexes. In the case of the ER, this is the cis-SNARE vesicle fusion complex (of which SYP-72 is a component). In this model, ERES and ERIS are in close proximity in a domain of the ER whose size approximates the diameter of an overlying Golgi stack. Golgi stacks are in tight association with the ER via a joint scaffolding matrix of tethering factors. It is proposed that as Golgi stacks move, they capture individual COPII vesicles released from ERES, and simultaneously release COPI vesicles. Both types of vesicles accumulate in the restricted ER–Golgi interface. When the Golgi stacks temporarily stop moving (docking phase), fusion of COPI and COPII vesicles to their respective target membranes takes place. Adapted from Lerich et al. (2012) Figure 13A.

The “Secretory Unit Concept” (daSilva et al., 2004) was therefore formulated as a consequence of the colocalization of Golgi and COPII markers. It describes a specific domain of the ER, containing ERES, which together with an overlying Golgi stack, constitutes a mobile secretory aggregate (see “Brandizzi” section in Robinson et al., 2015, for a discussion; see also Figure 6). This is a distinguishing feature of the endomembrane system of plant cells, since the Golgi apparatus in animal cells is usually seen as a fused ribbon of stacks in a perinuclear position that is nonmotile (Saraste and Prydz, 2019).

There are still many unknowns concerning the function of the secretory unit. For example, there is an ongoing discussion as to whether the mediators of bidirectional protein traffic between the ER and cis-Golgi are bona fide vesicles (COPI and COPII), or whether transport is accomplished through direct tubular connections that are partially coated with COPI or COPII proteins (see “Hawes” section in Robinson et al., 2015; Brandizzi, 2018: Robinson, 2020). It also remains unclear whether bidirectional transport is a continuous process or restricted to stationary Golgi stacks. What is clear, through the use of optical tweezers, is that the secretory unit is an extremely stable complex. This stability is not indicative of direct connections between the ER and Golgi membranes, but rather is due to a tethering complex based on the golgin AtCASP (Osterrieder et al., 2017).

COPI-mediated retrograde trafficking has been well-studied in animal cells and involves an interaction of γ-COP with the cytoplasmic tails of p24 dimers (Béthune and Wieland, 2018). There are also interactions between α- and β′-COP and dilysine motifs on retrograde cargo molecules such as the KDEL receptor. Plants also possess p24 proteins, and it is generally accepted that KDEL-receptor–ligand complexes destined for retrieval to the ER interact with both the COPI GTPASE ARF1 and p24 proteins (Pastor-Cantizano et al., 2016). The receptor for KDEL-ligands is known as ERD2 in yeast and plants and has a putative COPI dilysine cluster. It is thought that ERD2 cycles between the ER and the cis-Golgi, releasing ligands into the lumen of the ER after COPI-vesicle fusion due to the neutral pH in this compartment. After having returned to the cis-Golgi via COPII-vesicles, the low pH in the cis-cisternae causes ERD2 to bind KDEL ligands and also leads to a conformational change in the ERD2 receptor that exposes lysine residues, thereby creating a COPI-binding motif (Robinson and Aniento, 2020).

Although it cycles between the cis-Golgi and the ER, ERD2 curiously locates predominantly to the cis-Golgi, being detected in the lumen of the ER only after over-expression of KDEL-ligands. In a model proposed by Robinson and Aniento (2020), it is assumed that under normal conditions, ERD2 is rapidly exported out of the ER. It is therefore only transitorily present in the ER and is restricted to a disc-like domain immediately underneath the first cis-Golgi cisterna. Its presence there makes it difficult to distinguish from the cis-Golgi population of ERD2 in face views of the Golgi under a confocal laser-scanning microscope.

The scenario just described implies that ER import sites are closely associated with ERES and lie immediately beneath the cis-Golgi. That this is indeed so comes from studies to pinpoint ER-located tethering factors and SNAREs responsible for COPI-vesicle tethering and subsequent fusion, respectively (Lerich et al., 2012). An (X)FP-tagged plant homolog to TIP20 of the yeast Dsl1 COPI-tethering complex perfectly colocalizes with Golgi stacks irrespective of their motile status. However, in contrast, the Qc-SNARE SYP72, whose overexpression has no effect on ER–Golgi trafficking, only colocalizes with Golgi markers when the stacks are immobilized through actin depolymerization. These findings suggest that punctate SYP72-YFP signals on the ER, which do not colocalize with Golgi stack markers, represent predetermined docking sites, and that COPI-vesicle fusion with the ER is restricted to periods when Golgi stacks are stationary (see Figure 6; for details of the actual labeling, the reader is referred to Lerich et al., 2012). The most gratifying aspect of this publication is that the same strategy was employed most recently by to define “bidirectional transport portals” on the ER of the yeast *Pichia pastoris*: a rare case where a plant publication has provided a template for animal/yeast studies on vesicle-mediated protein transport.

## Andrew Staehelin: Elucidation of the nanoscale architecture of cells—highlights of a long journey

*Andrew Staehelin developed cutting-edge cryofixation methods that he used to characterize novel features of plant cell structures through electron microscopy. His work addresses cell plate formation during cytokinesis, thylakoid structure, as well as various other topics. Here he highlights early work on cellulose-synthesizing rosette complexes, and structure/function studies of the plant Golgi apparatus.*

The long-term goal of my career was to produce high-resolution 3D models of cellular organelles and cytoskeletal systems in their natural state. In turn, I hoped that this information would provide a structural framework for understanding molecular, biochemical, and fluorescent microscope data produced by other researchers.

As a graduate student in the 1960s, I recognized that the principal limitation of biological electron microscopy was not the resolution limit of the electron microscopes but the inability of researchers to preserve the native architecture of cells for viewing in an electron microscope. In particular, I recognized the limitations of chemical fixatives and that cryofixation provided the only alternative fixation method.

## The development of propane jet-freezing of turgid cells led to the discovery of novel membrane structures

The development of a propane jet-freezing apparatus capable of vitrifying up to 40-µm thick samples (described in Gilkey and Staehelin, 1986) yielded our first images of freeze-fractured vitrified cells. This enabled us to address the question of how cellulose fibrils are produced by plants and green algae capable of producing primary and secondary cell walls. Using growing semi-cells of the unicellular green alga *Micrasterias* as an experimental system, we demonstrated that cellulose fibrils were synthesized by plasma membrane rosette particle complexes (Giddings et al., 1980). Single rosettes were shown to produce ∼5-nm fibrils in primary walls, whereas rows of rosettes in arrays gave rise to larger diameter fibrils in secondary walls. These complexes were delivered in a preassembled form to the plasma membrane. More primitive algae possess linear particle complexes similar to those found in the bacterium *Acetobacter xylinum* (Brown and Montezinos,1976).

How does turgor pressure affect vesicle-mediated secretion and membrane recycling? Using sycamore, maple, and carrot suspension-cultured cells as experimental systems, we observed that during exocytosis, the vesicles collapsed into disc-shaped membrane appendages because their membranes were unable to be absorbed into the turgid plasma membrane (Staehelin and Chapman, 1987). Recycling of the appendage membranes involves the formation of horseshoe-shaped membrane infoldings that become cupped by closely associated ER membranes until the excess membrane has been resorbed, presumably by a lipid-hopping mechanism (Lev, 2010). The proteins were retrieved by CCVs.

## High-pressure freezing enabled us to develop functional, nanoscale models of ER, Golgi, and TGN cisternae

Thin section studies of high-pressure frozen Arabidopsis and tobacco root tip cells demonstrated that the Golgi stacks consisted of three types of structurally distinguishable cisternae, cis*-*, medial-, and trans-Golgi cisternae (Staehelin et al., 1990). These structural differences suggested that they were functionally different. We tested this hypothesis using antibodies against sugar epitopes of secreted polysaccharides and glycoproteins in cryofixed Golgi. The results confirmed our hypothesis. For example, antibodies against the backbone and the sidechains of xyloglucans bound only to trans-Golgi and TGN cisternae (Zhang and Staehelin, 1992), whereas with anti-native α-1,2 mannosidase antibodies, 90% of the label was in medial cisternae (Donohoe et al., 2013).

**Figure 7:**
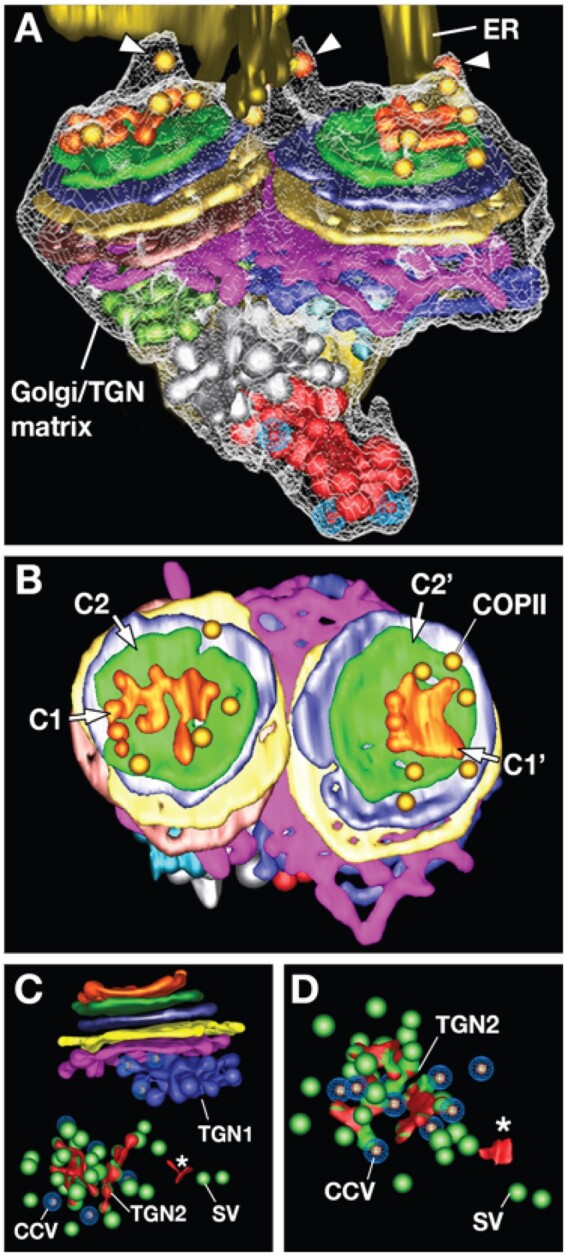
3D tomographic models of a dividing Arabidopsis Golgi stack and TGN cisternae. A, Interactions between the COPII scaffolds (arrowheads) and the cis*-*side of the Golgi/TGN matrix/scaffold connect ER and Golgi. The dividing Golgi possesses two sets of cis- and medial-cisternae (green, purple, and yellow) held together by the undivided trans-Golgi cisterna (pink). B, Face-on views of the cis*-*cisternae of the dividing Golgi shown in (A). The C1 and C1′ (orange) and the C2 and C2′ cisternae (green) are cisternal assembly intermediates. COPII vesicles are seen around the margins of these cisternae. C, 3D model images of a Golgi stack with a Golgi-associated TGN (TGN1) and a free, fragmenting TGN (TGN2). C and D, Release of the secretory and CCVs and residual cisternal fragments (asterisk, red) occurs simultaneously.

Studies of the trafficking of fluorescently labeled Golgi stacks in living tobacco BY-2 cells led to the formulation of the “stop-and-go” model of Golgi stack trafficking along ER-associated actin filaments (Nebenführ et al., 1999). What causes moving Golgi stacks to stop? Where do they stop? The discovery of a ribosome-excluding and Golgi-TGN encompassing scaffold/matrix (Figure 7A) provided the first clue. This scaffold was shown to originate as an external layer on budding COPII vesicles at ER exit sites and to contain the Atp115 scaffolding protein (Kang and Staehelin, 2008). Once assembled, this scaffold enabled budding COPII vesicles to capture passing Golgi by attaching to the cis side of the Golgi scaffold. After binding, the docked, wiggling Golgi pluck the COPII vesicles from the ER, which are then incorporated into growing cis cisternae. Simultaneously, the COPII scaffold molecules become integrated into the Golgi scaffold. These results led to the formulation of the revised “stop-pluck-and-go” model of Golgi trafficking (Staehelin and Kang, 2008).

Prior to the introduction of cryofixation to plant cell biology, electron microscopists searched in vain for COPII vesicles in chemically fixed cells. After visualizing COPII vesicles in high-pressure frozen root tip cells (Craig and Staehelin, 1988), we focused our efforts on characterizing the distribution of COPII and retrograde COPI-type Golgi vesicles around Golgi stacks. This work led to the discovery of two subtypes of COPI vesicles, COPIa and COPIb (Donohoe et al., 2007). COPIa and COPII vesicles were seen exclusively between ER exit sites and the cis-cisternae of Golgi stacks, whereas the COPIb vesicles were observed only around the medial- and trans-Golgi and TGN cisternae. This suggested that COPIa vesicles were involved in membrane recycling between the cis-cisternae and the ER, and the COPIb vesicles in recycling between medial-, trans-Golgi, and TGN cisternae (Donohoe et al., 2007). Quantitative analysis of cisternal membrane recycling demonstrated furthermore that during the transformation of the trans-most Golgi cisterna to a TGN cisterna, between 30% and 35% of the membrane is recycled (Kang et al., 2011).

Two central predictions of the cisternal progression model of Golgi trafficking are that new Golgi cisternae are assembled on the cis-side of the stacks, while old cisternae are shed on the trans-side (Day et al., 2013). Electron tomography enabled us to follow cisternal assembly from the initial fusion of two COPII vesicles on the surface of the existing cis-most cisterna followed by the growth and maturation of a new cis-cisterna (Figure 7B; Donohoe et al., 2013). In turn, the trans-most Golgi cisternae were observed to peel off the stacks, mature into TGN cisternae, and release the secretory and CCVs by cisternal fragmentation (Figure 7, C and D; Kang et al., 2011).

Further analysis of the de novo assembly of the cis-Golgi cisternae demonstrated that cis-cisternae are strictly cisternal assembly compartments from which escaped ER proteins are returned to the ER via COPIa vesicles. Activation of the enzymatic activities occurs only in the medial cisternae (Donohoe et al., 2013).

Looking back, I never imagined that the decision to focus my career on the development of improved cryofixation and specimen preparation methods would lead to such a wealth of novel insights into plant cell structures and functions. During this pursuit, I was astonished that it took 15 years for the electron microscopy research community to accept the high-pressure freezing technique. Now cryofixation is considered an essential tool for producing nanoscale 3D reconstructions of cellular membrane and cytoskeletal systems by means of electron tomography.

## Concluding remarks

As illustrated by the retrospectives in this review, the plant cell biology field has seen tremendous progress in the past decades, truly a bountiful harvest. In part, investigations are inspired by the unique nature of walled plant cells compared to other multicellular eukaryotes. In those cases, we have learned how plants have solved problems toward developing multicellular bodies differently from animals of fungi. In other cases though, plant cells have proven to be superior model systems for general eukaryotic cellular processes. The “vignettes” in this review show how deep investigation, along with implementing or developing new experimental approaches, can illuminate fundamental properties of plant cells, with impacts reaching to all fields of plant science. We hope these stories will continue to inspire future investigations and breakthroughs in the exciting field of plant cell biology, where so much is yet to be learned.

## Notes

All contributing authors conceived and wrote their individual part, as marked in each section, prepared figures and commented on a complete draft, and prepared and compiled by D.W.

The author responsible for distribution of materials integral to the findings presented in this article in accordance with the policy described in the Instructions for Authors (https://academic.oup.com/plcell) is: Dolf Weijers (dolf.weijers@wur.nl).
